# Unravelling the importance of the eukaryotic and bacterial communities and their relationship with *Legionella* spp. ecology in cooling towers: a complex network

**DOI:** 10.1186/s40168-020-00926-6

**Published:** 2020-11-12

**Authors:** Kiran Paranjape, Émilie Bédard, Deeksha Shetty, Mengqi Hu, Fiona Chan Pak Choon, Michèle Prévost, Sébastien P. Faucher

**Affiliations:** 1 Department of Natural Resource Sciences, Faculty of Agricultural and Environmental Sciences, McGill University, Sainte-Anne-de-Bellevue, QC Canada; 2 Department of Civil Engineering, Polytechnique Montreal, Montréal, QC Canada

**Keywords:** *18S rRNA* gene amplicon sequencing, Eukaryotic community, *Legionella pneumophila*, *Brevundimonas* sp., Dissolved organic carbon, Network analysis, Whole genome sequencing

## Abstract

**Background:**

Cooling towers are a major source of large community-associated outbreaks of Legionnaires’ disease, a severe pneumonia. This disease is contracted when inhaling aerosols that are contaminated with bacteria from the genus *Legionella*, most importantly *Legionella pneumophila*. How cooling towers support the growth of this bacterium is still not well understood. As *Legionella* species are intracellular parasites of protozoa, it is assumed that protozoan community in cooling towers play an important role in *Legionella* ecology and outbreaks. However, the exact mechanism of how the eukaryotic community contributes to *Legionella* ecology is still unclear. Therefore, we used *18S rRNA* gene amplicon sequencing to characterize the eukaryotic communities of 18 different cooling towers. The data from the eukaryotic community was then analysed with the bacterial community of the same towers in order to understand how each community could affect *Legionella* spp. ecology in cooling towers.

**Results:**

We identified several microbial groups in the cooling tower ecosystem associated with *Legionella* spp*.* that suggest the presence of a microbial loop in these systems. Dissolved organic carbon was shown to be a major factor in shaping the eukaryotic community and may be an important factor for *Legionella* ecology. Network analysis, based on co-occurrence, revealed that *Legionella* was correlated with a number of different organisms. Out of these, the bacterial genus *Brevundimonas* and the ciliate class *Oligohymenophorea* were shown, through in vitro experiments, to stimulate the growth of *L*. *pneumophila* through direct and indirect mechanisms.

**Conclusion:**

Our results suggest that *Legionella* ecology depends on the host community, including ciliates and on several groups of organisms that contribute to its survival and growth in the cooling tower ecosystem. These findings further support the idea that some cooling tower microbiomes may promote the survival and growth of *Legionella* better than others.

Video Abstract

## Background

Cooling towers are not typically thought of as ecological niches for microorganisms; yet, they harbour a vast quantity of microorganisms [1, 2]. A perfect example of their suitability as an ecological niche is that cooling towers are an important source of large community associated outbreaks of Legionnaires’ disease (LD), a severe bacterial pneumonia caused by several bacterial species of the genus *Legionella*, such as *Legionella pneumophila* [3–5]. Recent outbreaks due to cooling towers have been making headlines in North America, such as the 2015 New York City outbreaks (138 cases, 18 deaths) and the Disneyland outbreak in 2017 (12 reported cases) [6, 7]. Furthermore, cooling towers are also an important source of sporadic cases of LD [8, 9]. A study from 1978 to 1986, in the city of Glasgow, Scotland, revealed that around 28% of sporadic LD cases were associated with cooling towers [8]. The distance from the cooling tower is also believed to be an important risk factor [8, 9]. Due to their design, cooling towers produce high quantities of aerosols, which when contaminated with *L*. *pneumophila*, can spread the bacterium to the surrounding environment, reportedly as far as 12 km [10]. Individuals in the dispersion area inhaling the aerosols are at risk of infection, with risk increasing as the distance from the source decreases [9].

The Centre for Disease Control in the USA and the European Centre for Disease Prevention and Control have both reported increasing trends of LD in recent years [11–14]. For instance, the rate of incidence of LD in the USA increased from 0.42 cases per 100,000, in 2000, to 1.89 cases per 100,000, in 2015 (4.5-fold increase) [14, 15]. In the European Union, the incidence of reported cases increased by 50% from 2013 to 2017, with a reported incidence of 1.2 per 100,000, in 2013, to an incidence of 1.8 per 100,000, in 2017 [11]. The exact minimum infectious dose of *L*. *pneumophila* is currently a subject of debate. Quantitative microbial risk assessment models have been used to predict the risk of pneumonia associated with aerosolization of *Legionella* from different engineered water systems [16, 17]. In an outbreak associated with whirlpool spas, the model showed that an estimated dose of 35 CFU of *Legionella* may pose a severe clinical risk [17, 18]. Even with such a low infectious dose, it is clear that the *L*. *pneumophila* population must increase within a cooling tower to a high-enough threshold, so that sufficient contaminated aerosols are emitted to reach neighbouring population and cause illness. Consequently, understanding growth factors and ecology within cooling towers is of vital importance.

*Legionella* spp*.* are intracellular parasites of various protozoan species, such as amoebae and ciliates, and require these host for growth in water systems [19–22]. These host species are microbial grazers that feed on microbial communities. *Legionella* species have taken advantage of this trait by allowing their phagocytosis and then creating a suitable environment for replication within the phagosome of the host cell. This is achieved through the use of a type IV secretion system that translocates many effector proteins into the intracellular host environment [23]. More than 18,000 effector proteins are present in the pangenome of *Legionella* [24]. Different species or strain of *Legionella* can infect different host species, depending on the type of effector protein present in the genome [24]. In addition, host cells provide a safe means of transportation and protection from harsh chemicals, such as disinfectants found in the cooling tower environment, as some protozoa host can produce cysts [25–27]. The number of known host species is quite expansive, spanning several distant phylogenetic groups. For instance, *Acanthamoeba castellanii*, *Naegleria fowleri*, *Tetrahymena pyriformis* and human macrophages are host species belonging to different eukaryotic groups (respectively *Amoebozoa*, *Heterolobosea*, *Ciliophora*, *Chordata*) and are routinely used as host models for research [28]. Consequently, host diversity and abundance, and the factors influencing them in the cooling tower environment is an important aspect for *Legionella* survival, proliferation and transmission to humans.

Protozoan host species inhabit various engineered water systems, including cooling towers [29–31]. In these systems, the protozoa usually feed by grazing on a diversity of prey from the bacterial, algal, fungal and other protozoan communities [32, 33]. Several factors can influence the health and proliferation of the protozoan community. Firstly, the species and abundance of the prey community is an important factor for growth of the protozoan host population. Grazing is usually a selective process that depends on the prey species, prey morphology, prey size and physiological state of the prey and of the predator [34–36]. As a result, factors affecting the prey community will have consequences on the *Legionella* host community, and therefore, indirectly on the *Legionella* community. Accordingly, competition between prey species and non-prey species, competition by predation for the same prey by the non-*Legionella* host community and chemical and physical parameters of the environments are all elements that can have negative effects on the prey community [37–39]. For instance, *Bdellovibrio* spp*.* are bacterial parasites of different bacterial species that could potentially reduce the prey community [40]. Secondly, factors directly affecting the growth and survival of the host community may also affect the *Legionella* community. Predation by other eukaryotes, parasitism by the bacterial and viral communities and amensal relations with different organisms are direct biological interactions that could negatively affect the host community. For instance, *Pseudomonas aeruginosa* is known to kill the host amoeba *Acanthamoeba castellanii* using a type III secretion system and several toxins [41]. On the other hand, symbiotic and mutualistic relations can positively affect the host community. For example, certain species of *Parachlamydia* and *Candidatus* Procabacter are known endosymbionts of certain species of *Acanthamoeba* [42]. Consequently, the proliferation of the host population is likely dependent on a network of microbial interactions between the members of the bacterial and the eukaryotic communities. The host community is also affected by the physicochemical parameters of the environment, such as temperature and chlorine concentrations [30]. For instance, higher concentrations of chlorine are negatively correlated with the presence of protozoa in cooling towers and a temperature higher than 50 °C is correlated with fewer protozoa counts in water distribution systems [30].

Consequently, the eukaryotic community plays a crucial role in *Legionella* ecology. This implies that groups of microorganisms affecting the eukaryotic community may have a crucial, but indirect, effect on *Legionella* ecology in cooling towers or other systems. It also suggests that some specific microbiomes may be more permissive to *Legionella* survival and growth than others. Indeed, microbiomes with high levels of species interacting positively with *Legionella* would be more permissive for *Legionella* growth. Conversely, *Legionella* would have low survival and growth in a system which microbiome contains mostly species interacting negatively with *Legionella.* So far, the interplay between these communities in the context of *Legionella* colonization, survival and proliferation in cooling towers is still not well understood.

Furthermore, little research has been done on the entirety of the eukaryotic community in cooling towers. A recent study by Tsao et al. examined the relationship between the protist and bacterial community of three cooling towers [31]. However, this study focused on the protozoan hosts, and did not examine the entirety of the eukaryotic community present in the towers. Previously, we characterized the bacterial communities of 18 cooling towers. Several potential interactions between bacteria and *Legionella* spp*.* and *L*. *pneumophila* were identified. The presence of *Legionella* was associated with the presence of several bacterial taxa, such as *Brevundimonas*, *Porphyrobacter* and *Xanthobacteraceae*, but negatively correlated with *Pseudomonas* [2]*.* Consequently, we hypothesize that the presence of specific microbial groups could increase permissiveness of the microbiome of cooling towers to *L*. *pneumophila* colonization, survival and proliferation through their interactions with *L*. *pneumophila*’s host species. Our objective was to profile the eukaryotic communities of the same 18 cooling towers using an *18S rRNA* amplicon sequencing approach. The relationship between the eukaryotic community and the bacterial community was analysed in the context of *Legionella* ecology, along with the associated physicochemical characteristics of the cooling towers. A network analysis, based on co-occurrence, was performed between the eukaryotic community and the bacterial community to uncover potential interactions between members of the microbial community. The results from the network analysis lead us to investigate the interaction between a bacterial isolate of *Brevundimonas* sp*.*, *L*. *pneumophila* and ciliates hosts species. Furthermore, whole genome sequencing revealed potential mechanisms by which the *Brevundimonas* isolate favours the growth of *L*. *pneumophila*.

## Results

### Sequencing results

A total of 4,280,578 paired reads were generated from the MiSeq run. The Mothur MiSeq SOP was followed for processing the sequencing data [43]. Quality filtering, denoising and chimera removal of the raw sequences removed a total of 350,988 low-quality sequences, keeping 3,939,401 sequences. The resulting sequences were then classified using the Bayesian classifier implemented in Mothur and the Silva ribosomal RNA reference database [43–46]. From the classification, 1,891,109 sequences were identified as bacterial sequences, 1029 sequences as Archean and 260,506 sequences as “unknown”. Most bacterial sequences were identified as *Proteobacteria*, mainly *Gamma-* and *Alpha*-*proteobacteria.* The first three most abundant bacterial sequences were identified as an unclassified *Gammaproteobacteria* sequence, *Porphyrobacter* and an unclassified *Beijerinckiaceae* sequence. These three most abundant sequences constituted around 60% of the total bacterial sequences. The bacterial, archaeal and unknown sequences were removed from the data, leaving 1,786,757 eukaryotic sequences (Supplementary Table S1).

The eukaryotic sequences were then clustered into a total 44,183 different OTUs. The counts for each replicate, before and after using the Mothur pipeline, can be viewed in the Supplementary Table S1. For statistical analysis, the OTU counts of each cooling tower sample was created by averaging the OTU read counts of the three replicates. Microbiome analyst was then used for rarefying the data [47]. Before rarefaction, the averaged OTU counts ranged from 3861 counts to 105,815 counts for the different cooling tower samples (Supplementary Table S2). Good’s coverage estimator was used to evaluate if the sequencing depth was adequate for diversity analysis. The estimator averaged 97.01%, ranging from a minimum of 90.36% to a maximum of 99.76% depending on the sample. The data were filtered, rarefied and normalized to create an OTU table for analysis (see the “Materials and methods” section). This OTU table had 3484 read counts per sample (Supplementary Table S2). The Good’s coverage for this rarefied dataset averaged 97.65% ranging from 96.21 to 99.54% depending on the sample (Supplementary Table S2).

Furthermore, a blank sample was sequenced in order to determine the presence of any contaminating sequences coming from any steps of the sequencing library preparation used. This blank consisted in running a sterile filter, from the same lot used for the cooling tower samples, through the same DNA extraction protocol and *18S rRNA* DNA library protocol as mentioned in the “Materials and methods” section. After sequencing and processing the raw sequence data, the blank contained only 45 sequences, which could be clustered into a total of 15 OTUs. These OTUs were classified as “Unclassified *Embryophyta*” (plants), when using the Silva ribosomal reference database. This indicates that the cooling tower data were not contaminated by sequencing reads coming from elsewhere.

### Eukaryotic profile of cooling towers

**Fig. 1:**
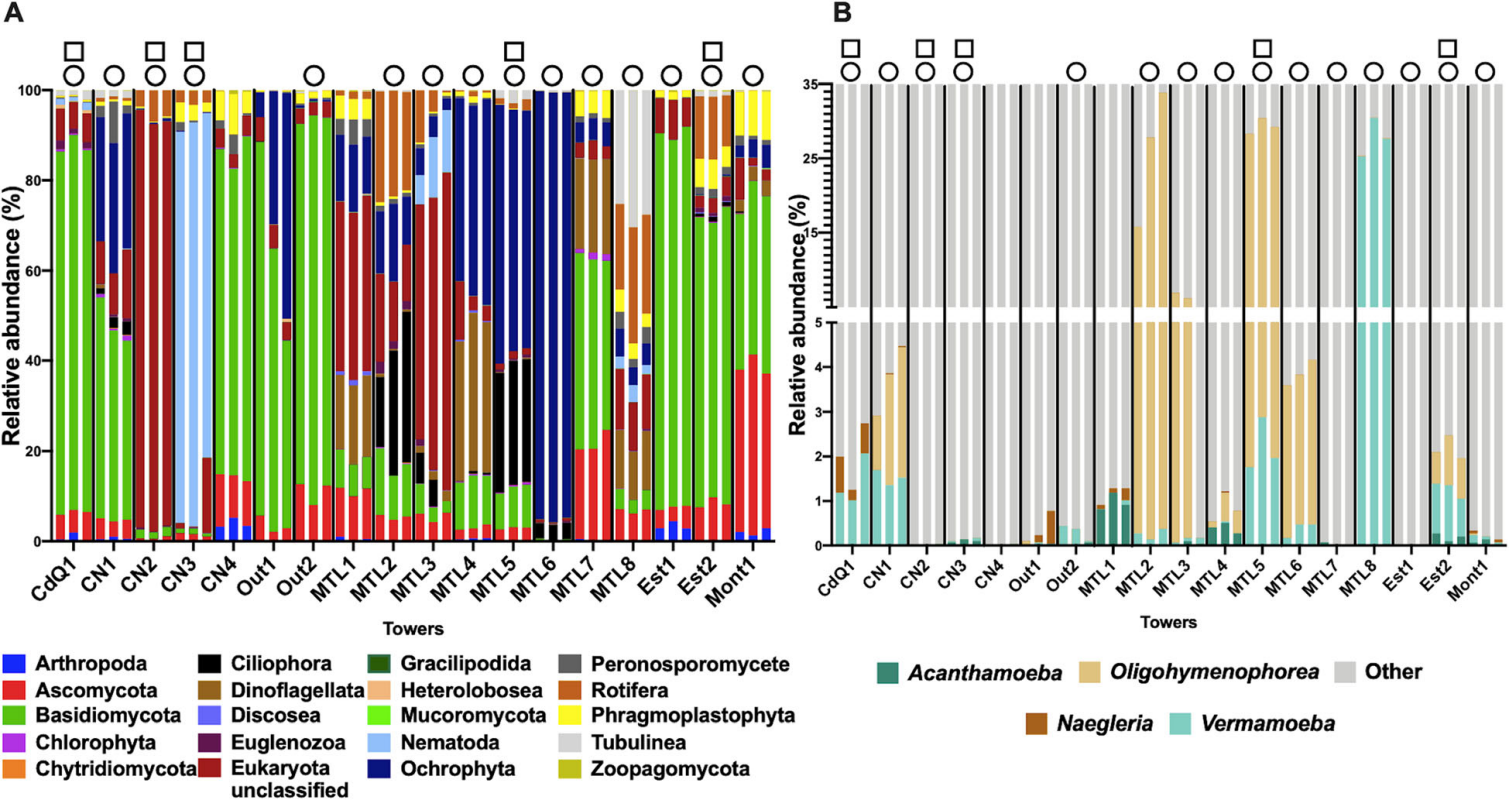
**a** Relative abundance of eukaryotic taxa present in cooling towers sampled in Quebec, Canada, classified at the class or phylum level. **b** Relative abundance of known host taxa of *L*. *pneumophila* and *Legionella* spp. in cooling tower samples. The group “others” represent the rest of the taxa present in the cooling towers. Individual replicates of each tower are shown. Circles represent towers in which *Legionella* spp*.* were detected (by *16S rRNA* gene amplicon sequencing), whereas squares represent towers contaminated with *L*. *pneumophila* (detected by qPCR), as previously published [2]

The *18S rRNA* gene targeted amplicon sequencing revealed a diverse community of eukaryotes inhabiting the cooling tower environment (Fig. 1a). The characteristics of each tower can be seen in Supplementary Table S3. Overall, the community could be divided into 20 different phyla and classes. Fungal groups were the most abundant and prevalent taxa in the cooling tower samples, with the *Basidiomycota* and *Ascomycota* classes being the most dominant (Fig. 1a). For instance, the *Basidiomycota* class dominated (more than 50 % of the community) the eukaryotic community in eight out of the 18 towers (Fig. 1a). Several other fungal groups, such as *Zoopagomycota* (*Zygomycota*), *Chytridiomycota* and *Mucoromycota*, were detected but at abundances of less than 1% across all towers sampled. Several taxa comprising known photosynthetic organisms were also detected, such as the *Chlorophyta* (Microalgae), *Dinoflagellates* and *Ochrophyta*. Towers MTL4, MTL5 and MTL6 contained notably high numbers of *Ochrophyta*. Additionally, micro-animals belonging to the *Nematoda* and *Rotifera* taxa were identified in many samples, such as in tower CN3 where nematodes constituted over 80% of the eukaryotic population. Free-living nematodes are important but underappreciated players in freshwater sediments ecology [48] and could potentially also play an important role in cooling towers. Nevertheless, this high abundance is likely due to their multicellular nature. Macro-eukaryotes were also identified with towers containing sequences related to insects (*Arthropoda*) and plants (*Phragmoplastophyta*). For instance, plant-related sequences reached around 10% of the community in tower Mont1.

Interestingly, several taxa harbouring known host species of *L*. *pneumophila* were also present in the towers, including *Ciliophora*, *Discosea*, *Heterolobosea*, *Nematoda* and *Tubulinea* (Fig. 1a) [28]. Out of these taxa, we examined the distribution of four of the most important host taxa: the *Acanthamoeba* genus (*Discosea*), the *Vermamoeba* genus (*Tubulinea*), the *Naegleria* genus (*Heterolobosea*) and the *Oligohymenophorea* class (*Ciliophorea*) (Fig. 1b). These four taxa contain well-established host cell species, such as *Acanthamoeba castellanii*, *Vermamoeba vermiformis*, *Naegleria fowleri* and *Tetrahymena pyriformis*, respectively [28]. The *Nematoda* class was not included as a potential host taxon, as it is still unclear whether nematodes actually promote growth or simply ensure survival of *L*. *pneumophila* [49, 50]. Since we could, at most, only resolve the OTUs to the genus levels, these groups represent potential hosts of *Legionella* species, since not all species of these groups may be permissive host cells. The relative abundance of host taxa was less than 5% in most towers (Fig. 1b); however, the host read counts reached a relative abundance of around 30% for three towers, MTL2, MTL5 and MTL8 (Fig. 1b).

### Alpha diversity and beta diversity are affected by dissolved organic carbon

**Fig. 2:**
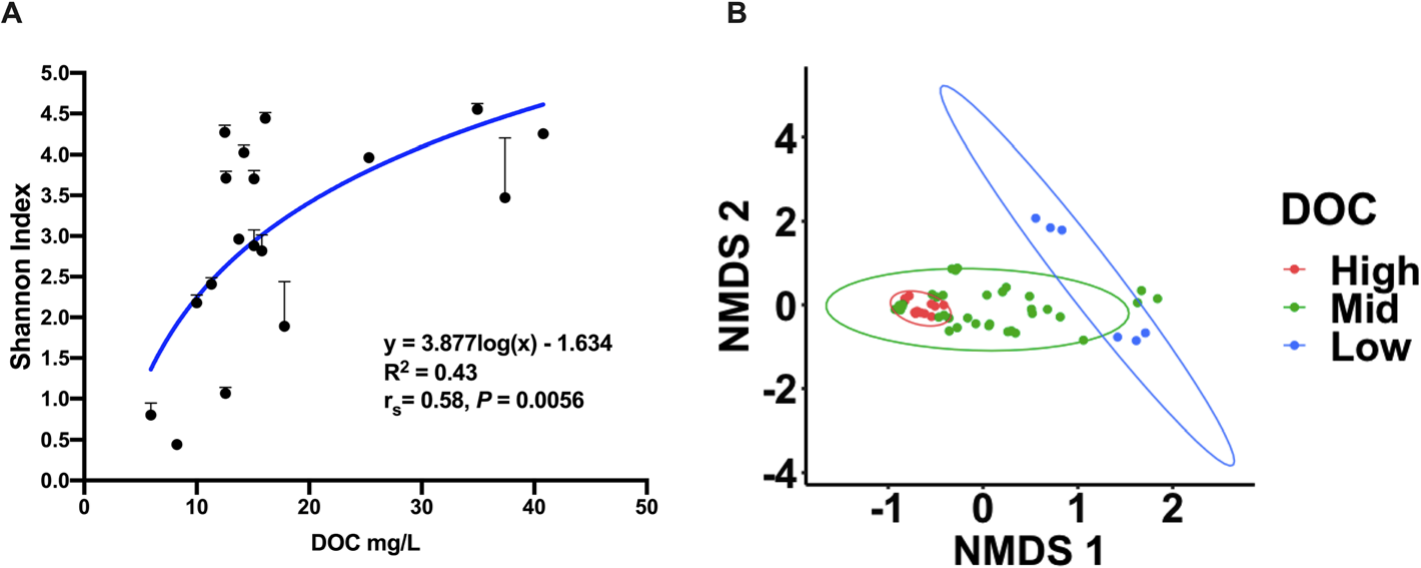
**a** Alpha diversity of cooling towers plotted against DOC levels of each tower. A semi-logarithmic curve fit the data best, using non-linear regression. The average and standard deviation of each tower is shown. **b** Non-metric multidimensional scaling plot of cooling towers eukaryotic communities categorized by DOC levels and using ANOSIM to evaluate statistical significance of dissimilarity between communities (*R* = 0.21, *P* = 0.118, stress = 0.102). The categories are as followed: < 10 mg/L of DOC were grouped as low; 10 to 20 mg/L were categorized as mid; 20 to 40 mg/L were categorized in the high group. The plot graphs the Bray-Curtis dissimilarity index of each replicate of each tower. Towers with high (red) and low levels (blue) of DOC clustered separately (*R* = 0.75, *P* = 0.0667; see Supplementary Figure S1)

Alpha diversity of towers was analysed using the Shannon index and the effect of physicochemical parameters was investigated. Statistical analysis was conducted on the averaged results of the three replicates of each cooling tower sample. Overall, dissolved organic carbon levels (DOC) were positively correlated with alpha diversity (Fig. 2a); however, the correlation between DOC and alpha diversity was modest (spearman’s *r*_s_ = 0.58, *P* = 0.0056), with DOC following a non-linear regression model (*R*^2^ = 0.43).

Beta diversity was calculated with the Bray-Curtis dissimilarity index and visualized using non-metric multidimensional scaling plot (NMDS). ANOSIM was used to determine statistical significance and dissimilarity between communities. The beta diversity analysis revealed that DOC levels could partially explain the clustering of the cooling tower communities when using NMDS (Fig. 2b). Thus, communities that had high and low levels of DOC formed distinct clusters. Conversely, towers with medium levels of DOC shared similarity with the other two groups. When comparing only the high and low DOC towers, ANOSIM revealed high dissimilarity between these two groups, with an *R* value of 0.75 (Supplementary Figure S1). However, the *P* value was around 0.07 indicating that the two groups were not statistically different from one another.

**Fig. 3:**
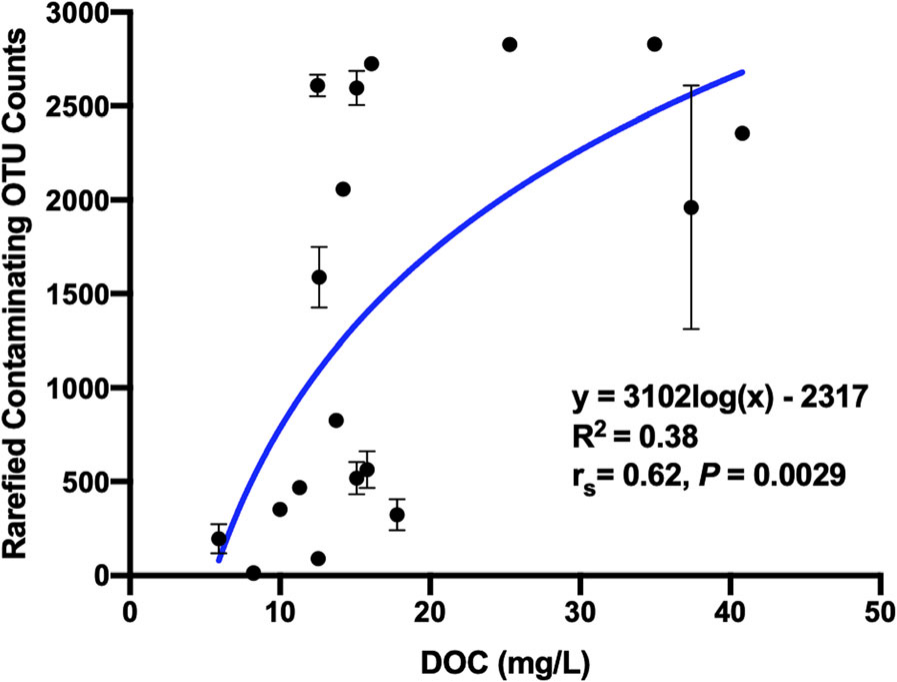
Rarefied contaminating OTU counts of tower as a function of the respective DOC levels for all sampled cooling towers. The average and standard deviation of each tower is shown. Non-linear regression was used to fit a semi-log curve to the data

We hypothesized that a substantial amount of DOC in cooling towers comes from biological contaminants in the air, such as spores and pollen. These contaminants are likely captured by the water droplets and spread in the cooling tower environment. To investigate this, we grouped OTUs likely to produce spores, pollen or seeds, as well as OTUs comprising airborne insects, and plotted them as a function of DOC levels for each tower. This group was named “contaminating OTUs”, as these OTUs are mostly comprised of organisms not found in water systems and likely originating from other types of ecosystems, such as green spaces. Consequently, “contaminating” OTUs were the sum of OTUs assigned to the taxa *Basidiomycota*, *Ascomycota*, *Arthropoda* and *Phragmoplastophyta*, after rarefying the dataset. Interestingly, a modest positive correlation (Spearman *r*_s_ = 0.62, *P* = 0.003) was observed between DOC levels and relative abundance of contaminating OTUs (Fig. 3). Using non-linear regression, the data followed a semi logarithmic curve (*R*^2^ = 0.38; Fig. 3). Thus, contaminating OTUs seem to contribute to DOC.

### Network analysis

**Fig. 4:**
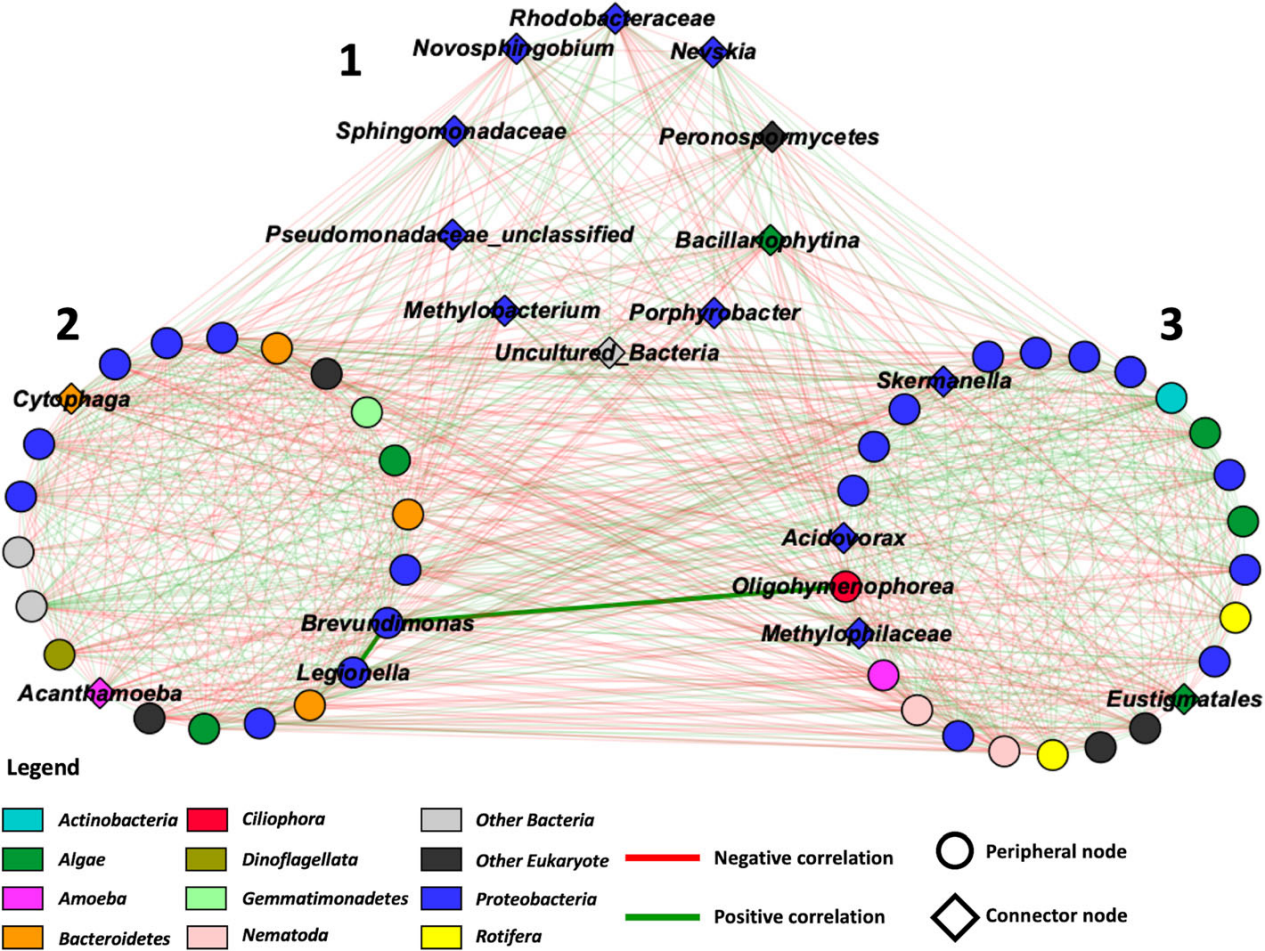
Microbial ecological network showing correlated taxa (Bacteria and Eukaryotes) in cooling tower samples organised into modules (1 to 3). Green edges represent positive correlations between taxa, and red edges represent negative correlations between taxa. Peripheral and connector nodes are respectively represented by circles and diamonds. A positive correlation can be observed between *Brevundimonas*, *Oligohymenophorea* and *Legionella*, indicated by thicker edges

Next, putative ecological relationships between the different taxa of the microbial community of the cooling towers were identified by constructing a microbial ecological network based on co-occurrence (Pearson’s correlation) using the MENA pipeline and visualized using Cytoscape 3.7.1 (Fig. 4) [51, 52]. The network was constructed from our previously published bacterial profiling dataset (see [2]) and the eukaryotic community profiles. However, to reduce the number of ecologically non-relevant interactions, the contaminating OTUs were removed from the dataset prior to rarefaction for this analysis. As a result, some of the towers were left with less than 75% of the original sequence counts. Those towers were therefore not considered for the network analysis.

Overall, the network was constituted of 58 nodes and 851 edges (Fig. 4). The general properties of the network revealed that the network did not have scale free or small world properties; however, it showed low modularity (*M* = 0.128) with the presence of 3 modules (Fig. 4) [51]. The genus *Legionella* could be found in module 2 along with *Brevundimonas and Acanthamoeba*. *Oligohymenophorea*, another host taxon of *Legionella*, was identified in module 3.

To understand the ecological roles of the taxa that constituted each module, the nodes were classified by their within-module (*Z*_i_) and among-module (*P*_*i*_) connectivity into peripheral nodes or connector nodes [51, 53]. The data can be visualized in Fig. 4. Peripheral nodes reveal a specialist ecological behaviour, whereas connector nodes indicate a more generalist ecological behaviour [51]. Module 1 consisted of ten connector nodes. These were *Rhodobacteraceae*, *Novosphingobium*, *Sphingomonadaceae*, unclassified *Pseudomonadaceae*, *Methylobacterium*, uncultured bacteria groups, *Porphyrobacter*, *Bacillariophytina*, *Peronosporomycetes* and *Nevskia*. *Cytophaga* and *Acanthamoeba* were the only connector nodes of module 2 (containing *Legionella*). Finally, four connector nodes were found in module 3. These were *Acidovorax*, *Methylophilaceae*, *Eustigmatales* and *Skermanella.*

**Fig. 5:**
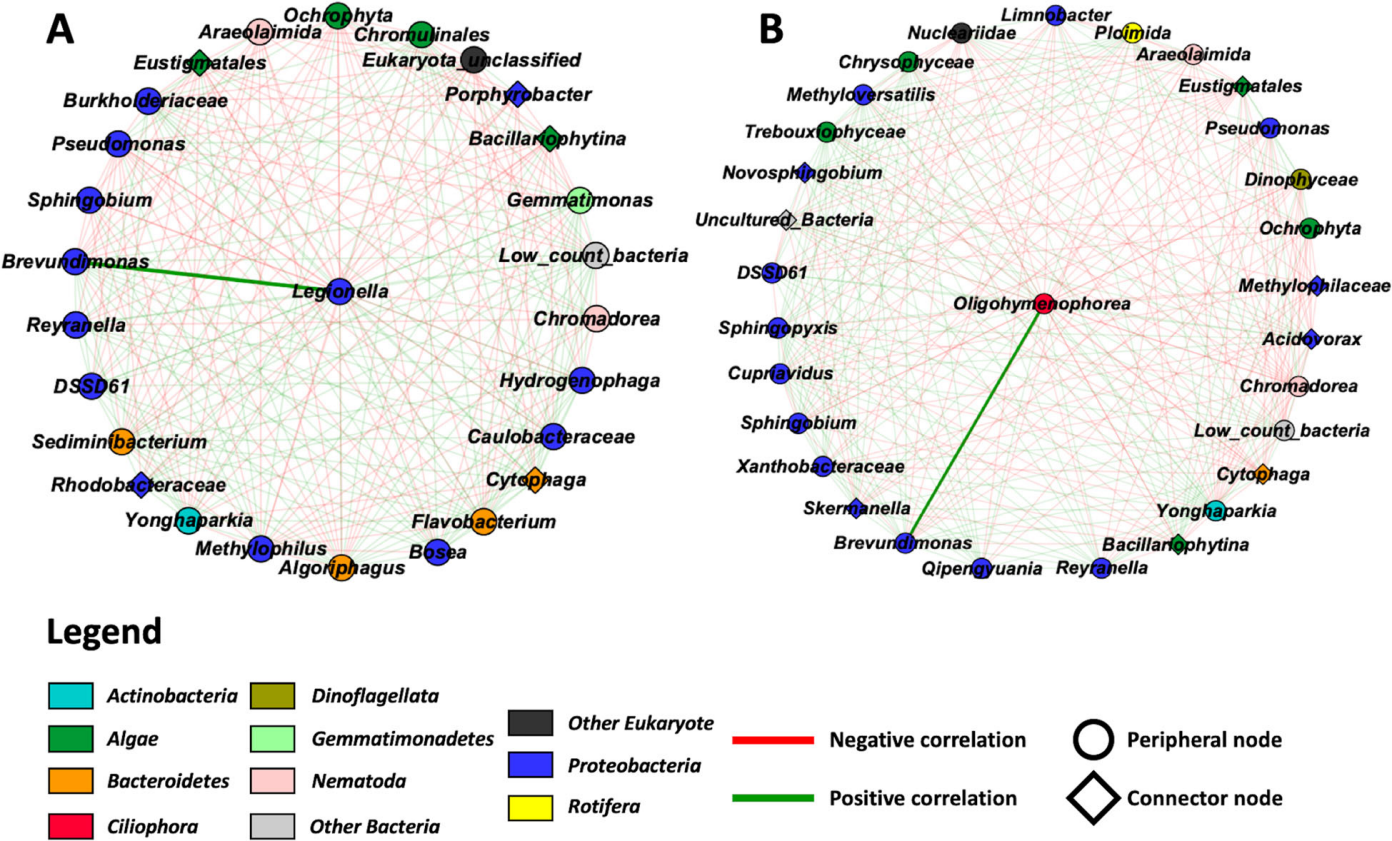
Sub-network showing first neighbour taxa of *Legionella* (**a**) and *Oligohymenophorea* (**b**). Green edges represent positive correlations between taxa and red edges represent negative correlations. The diamonds and circles represent connector nodes and peripheral nodes, respectively.

*Legionella* was classified as a peripheral node within module 2 of the network (Fig. 4). In order to better visualize *Legionella*’s position and role within the network, a sub-network was constructed using the first neighbour nodes of *Legionella* (Fig. 5a). This sub-network revealed that *Legionella* was directly correlated with 26 different taxa. The *Legionella* neighbours were diverse, including *Actinobacteria*, *Bacteroidetes*, *Gemmatimonadetes*, *Nematoda*, *Protebacteria* and algae such as *Ochrophyta*. Several of these bacterial neighbours were previously identified, using LEfSe, as predictors of varying levels of *Legionella* in the towers [2]. Finally, several neighbours were connectors nodes, such as *Bacillariophytina* (diatoms), *Cytophaga*, *Eustigmatales*, *Porphyrobacter* and *Rhodobacteraceae*. Of note, *Cytophaga* is the node with the most connection in the network with a total of 41 connections.

### Bacterial predictors of *Oligohymenophorea*

Eukaryotic profiling revealed that several *Legionella* host taxa were identified in the tower samples and that some of these taxa were positively correlated with *Legionella*. Indeed, *Oligohymenophorea* counts and *Vermamoeba* counts correlated positively with *Legionella* counts (Spearman’s *r*_s_ = 0.72, *P* = 0.0007 and Spearman’s *r*_s_ = 0.6, *P* = 0.005, respectively). *Oligohymenophorea* is a class of ciliates containing known host species of *L*. *pneumophila*, such as *Tetrahymena pyriformis* [21]. Since ciliates are microbial grazers and important in the ecology of *Legionella*, we sought to identify possible preys of *Oligohymenophorea*. First, we constructed a sub-network consisting of the first neighbours of *Oligohymenophorea* (Fig. 5b). Next, we performed a LEfSe analysis on our bacterial dataset to identify bacterial taxa that could predict the presence of *Oligohymenophorea* in the cooling towers [54]. To this end, we categorized the towers based on the number of rarefied read counts classified as *Oligohymenophorea* from our eukaryotic dataset. Thus, three groups were created: high (> 100 counts), low (1 to 100 counts) and absent (0 counts).

**Fig. 6:**
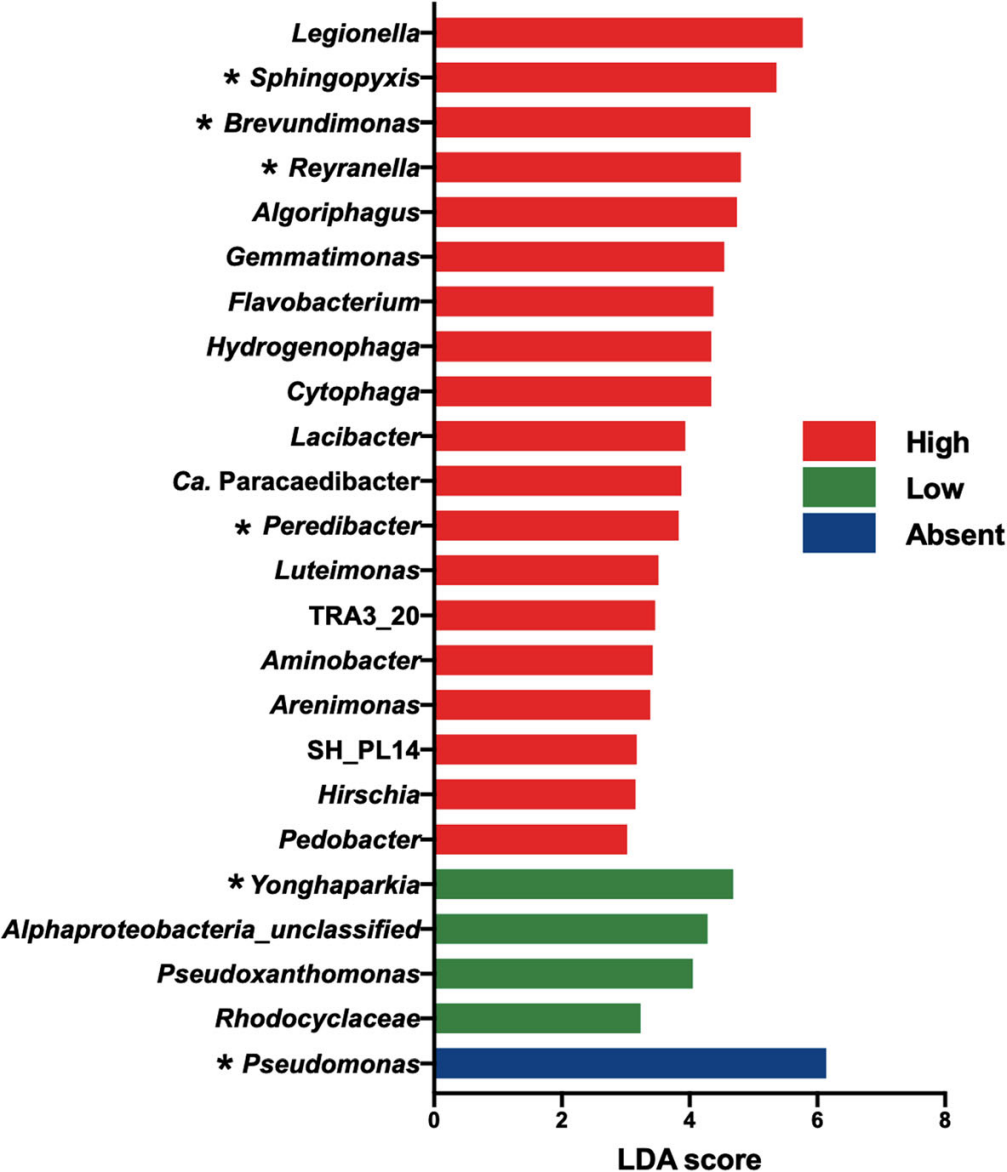
Bacterial taxa predicting towers containing varying levels of *Oligohymenophorea* using LEfSe. The towers were classified according to the number of sequences assigned to the *Oligohymenophorea* class: absent (0 count, blue), low (between 1 and 100, green), high (more than 100, red). Taxa previously identified as predictors of *Legionella* are indicated with “*” [2]

Bacterial predictors could be identified for all three *Oligohymenophorea* level categories (Fig. 6): 19 bacterial taxa were predictive of high levels, four taxa were predictive of low levels, and one genus was predictive of an absence of *Oligohymenophorea* (Fig. 6)*. Legionella* was the most predictive genus for a high level of the ciliates, whereas *Pseudomonas* was predictive of an absence of ciliates in the towers. *Brevundimonas* was also predictive of high levels. Several species were identified by both the network analysis and LEfSe, such as *Brevundimonas*, *Rayranella* and *Sphingopyxis* (Figs. 5b and 6). Moreover, some bacterial predictors of *Olygohymenophorea* were previously found to be predictors of the presence of *Legionella* spp. [2]. For instance, *Yonghaparkia*, *Reyranella*, *Brevundimonas* and *Sphingopyxis* were predictive of towers containing *Legionella* [2]. Conversely, *Pseudomonas*, which correlated negatively with *Oligohymenophorea* (Fig. 5b), was predictive of towers that did not have *Legionella* [2]. Finally, several bacterial predictors were also identified as direct neighbours of *Legionella* or in the same module as *Legionella* in the network (Figs. 4 and 5), for instance, *Pseudomonas*, *Brevundimonas*, *Gemmatimonas*, *Cytophaga*, *Flavobacterium* and *Reyranella*.

### The bacterial predictor *Brevundimonas* is a prey for *Oligohymenophorea* host cells

The LEfSe analysis revealed that the genus *Brevundimonas* is a predictor of towers with high levels of *Oligohymenophorea*. We hypothesized that this correlation between *Brevundimonas* and *Oligohymenophorea*, also seen with the network analysis, is probably due to a prey-predator relationship. *Brevundimonas* SPF441 was isolated from a cooling tower and subjected to whole genome sequencing. The sequencing run generated a total of 500,485 paired reads between 35 and 301 nucleotides in length. After using Trimmomatic (see “Materials and methods” section), a total of 33,119 reads were removed, leaving 467,366 reads. Spades assembled the reads into 66 contigs with a total sequence length of 3,201,388 bp. The N50 was 98,818 bp, with the shortest contig at 238 bp and the longest at 344,975 bp. The median depth was calculated at 7.55X. Prokka identified 3161 coding sequences (CDS), 3 rRNA elements and 51 tRNA elements. A short description of the metabolic genes can be viewed in the Supplementary Document DS1. Analysis of the genome with MiGA revealed that our isolate is closely related to *Brevundimonas vesicularis* with a 95.5% average nucleotide identity [55]. The *16S* RDP classifier implemented in MiGA also showed that the isolate was classified within the *Brevundimonas* genus. These results indicate that the isolate is most likely a species within the *Brevundimonas* genus; however, identifying the species would require additional tests.

**Fig. 7:**
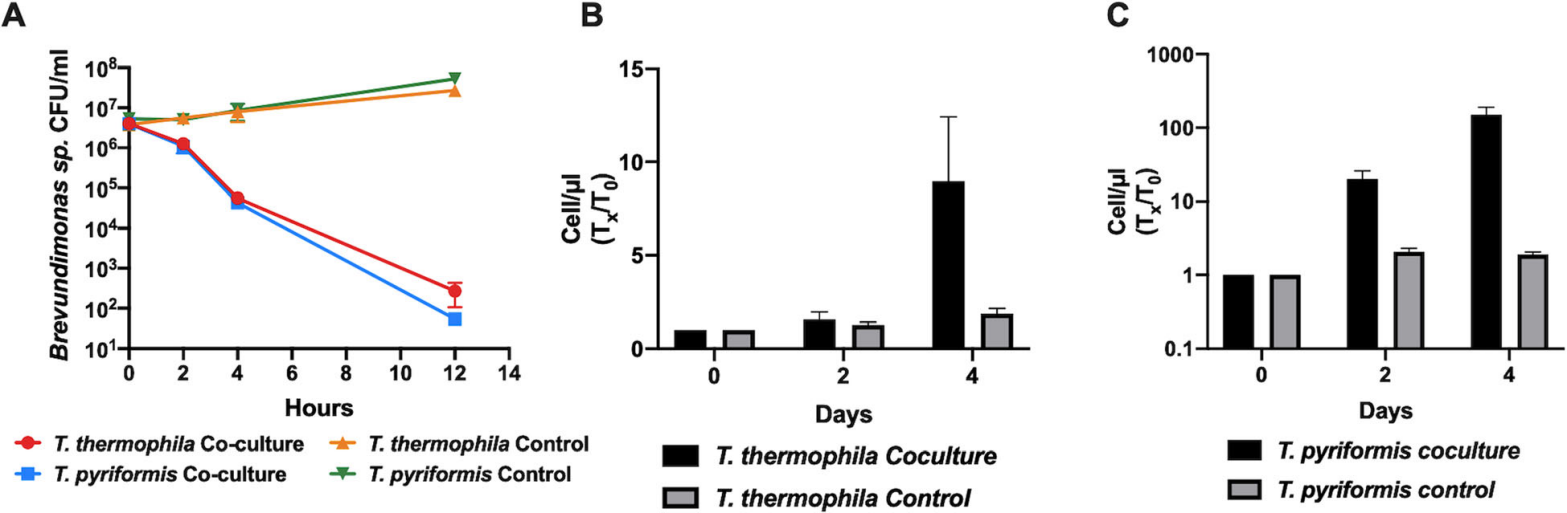
*Brevundimonas* SPF441 is a prey for *Tetrahymena*. Survival of *Brevundimonas* SPF441 when co-cultured with *T*. *thermophila* and *T*. *pyriformis*, in plate counting broth (**a**). *Brevundimonas* SPF441 suspended alone in plate counting broth was used as the control. Growth (*T*_x_/*T*_0_) of *T*. *thermophila* (**b**) and *T*. *pyriformis* (**c**) fed with *Brevundimonas* SPF441, in 10 mM Tris (pH 7.5) and incubated at 30 °C and 25 °C, respectively. As a control, the two ciliates species were incubated in 10 mM Tris without feeding of *Brevundimonas* SPF441 at the same temperatures mentioned above

Given that the network analysis revealed that the genus *Brevundimona*s was positively correlated with the genus *Legionella* and the class *Olygohymenophorea*, co-culture experiments were undertaken between the isolated *Brevundimonas* SPF441 and the ciliates *T*. *pyriformis* and *T*. *thermophila*. The two species of *Tetrahymena* tested are known host species for *L*. *pneumophila* and belong to the *Oligohymenophorea* class [21, 56]. The *Brevundimonas* SPF441 counts decreased by 5 logs after 12 h of co-culture with both *Tetrahymena* species. In contrast, no decrease in CFU numbers was seen when *Brevundimonas* SPF441 was incubated alone in the media (Fig. 7a, control). These drastic decreases suggest that *Brevundimonas* SPF441 is being consumed by *Tetrahymena*. However, certain *Tetrahymena* species are known to reject certain species of bacteria they consume by pelletizing them in packages and excreting them from their cells [57]. To test whether or not the *Brevundimonas* SPF441 cells were being consumed for nutrition, co-cultures where performed in Tris buffer, in which *Tetrahymena* is unable to grow. When fed with *Brevundimonas* SPF441, *T*. *thermophila* number increased by 9-fold and *T*. *pyriformis* number increased by 150-fold over 4 days (Fig. 7b, c). Minimal growth was observed for the ciliates in buffer alone. Our results confirm that this bacterium is readily consumed by the ciliates and is sufficient for growth of the ciliate population.

### *Brevundimonas* SPF441 promotes growth of *L*. *pneumophila*

**Fig. 8:**
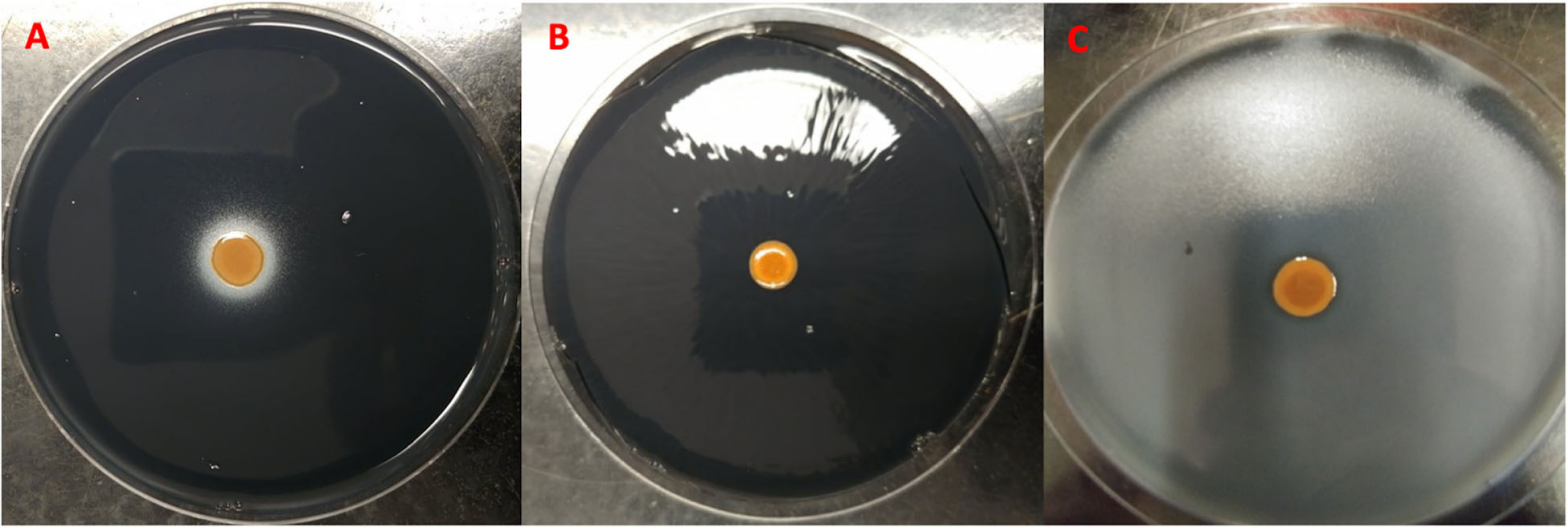
*Bevundimonas* SPF441 stimulates growth of *L*. *pneumophila*. Stimulation assay was carried on CYE agar without l-cysteine supplementation (**a**) or with l-cysteine supplementation (**c**). *L*. *pneumophila* was inoculated in soft agar which was poured on the surface of CYE plate (**a** and **c**). *Brevundimonas* SPF441 was spotted on each of those plates, and alone as control (**b**)

In addition, *Brevundimonas* could also directly promote the growth of *Legionella* in water systems. To investigate this possibility, a stimulation assay was performed based on the fact that *L*. *pneumophila* requires supplementation of l-cysteine to grow on CYE plates. The assay showed that *L*. *pneumophila* grew in a concentric circle around the colony of *Brevundimonas* SPF441 on plate lacking l*-*cysteine, which was visualized as a white halo (Fig. 8)*.* This white halo around *Brevundimonas* SPF441 was not seen on plates not inoculated with *L*. *pneumophila* (Fig. 8b). Furthermore, the white halo was confirmed to be *L*. *pneumophila* by re-streaking on CYE with l-cysteine (growth) and without l-cysteine (no growth)*.* These results indicate that *Brevundimonas* SPF441 was able to stimulate the growth of *L*. *pneumophila* on CYE plates without l-cysteine. Analysis of the genome of *Brevundimonas* SPF441 revealed several genes related to cysteine metabolism, such as cystathionine gamma-lyase and cysteine-*S*-conjugate beta-lyase (Supplementary Document DS1).

## Discussion

*Legionella* outbreaks are complex phenomena that are not well understood. The presence of protozoan-host species is crucial for the bacterium’s proliferation in the cooling tower environment [19–22]. Consequently, studying the ecology of the host community is key to providing insights into the mechanisms that may lead to high *Legionella* concentrations in cooling towers. In the present work, we characterized the eukaryotic communities of 18 cooling towers, using an *18S rRNA* gene amplicon sequencing approach. The eukaryotic community was analysed in relation to the bacterial and *Legionella* communities of these same towers, previously identified using a *16S rRNA* gene amplicon sequencing approach [2].

### Sequencing results

Although more than 4.2 million reads were sequenced from our library, around 60% of the data was removed for analysis. The initial denoising steps only removed 8% of the sequences due to poor quality or presence of chimeras. The amplification and sequencing of bacterial reads caused most of the data loss. Indeed, around 44% of the reads were classified as bacterial sequences belonging to *Proteobacteria*, such as *Porphyrobacter* and unclassified *Beijerinckiaceae*. After removal of the non-eukaryotic sequences, around 1.7 million sequences remained for analysis, constituting about 40% of the original data. The amplification of a high number of bacterial sequences suggests that the primers used are not specific for eukaryotic organisms. Subsequent to the acquisition of the sequencing data for this project, the Earth Microbiome Project warned that the EukBr reverse primer described in their *18S rRNA* protocol can indeed amplify bacterial sequences [58]. Additionally, no bacterial sequences were amplified when we ran a blank through the same pipeline. This indicates that the bacterial sequences were not amplified due to contamination from the kits. Despite the data lost, we estimate that our sequencing depth was adequate to perform subsequent analysis based on Good’s coverage (Supplementary Table S2) [31, 59].

### Microbial loop in cooling towers

The complexity and diversity of the ecosystems contained within the towers is evidenced by the presence of multiple trophic levels within the cooling tower environment. Indeed, the presence of primary producers (photoautotrophs, chemoautotrophs), microbial grazers (amoeba, ciliates, nematodes and rotifers) and several different functional bacterial groups (heterotrophic decomposers, perchlorate reducers, nitrogen fixers, chemolithotrophs) suggests the existence of a local microbial loop within these niches [60]. In this scenario, primary producers, such as the algae *Tribouxiophyceae*, *Ochrophyta*, *Eustigmatales*, *Chrysophyceae*, *Chromulinales* in module 2 and 3 or bacterial chemoautotrophs, release dissolved organic carbon (DOC) through waste products and dead cells. Primary production through photosynthesis may be possible as cooling towers are not closed systems, and most have openings that allow light to reach the basin or fill. The DOC is then consumed by the heterotrophic bacterial and fungal populations. Subsequently, the carbon travels up the trophic levels through different groups of microbial grazers (unicellular, such as amoeba and ciliates, then multicellular, such as nematodes and rotifers). The microbial grazers re-introduce the carbon into the cycle in the form of dead cells and waste products. This dynamic indicates that cooling towers not only allow survival of microorganisms but also act as sustainable and active breeding grounds for microorganisms, despite the use of disinfection strategies.

This local microbial loop may impact the *Legionella* community. Indeed, towers with higher levels of DOC, produced by primary production, may be able to support a higher population of chemoheterotrophic microorganisms. This in turn may promote the establishment of a *Legionella* host population, as more prey species (either primary producers or chemoheterotrophic microorganisms) may allow for more proliferation of host species. As a result, *Legionella* could grow more numerously in towers with higher levels of primary production. Moreover, previous research has shown that infected amoeba occur more frequently in cooling towers than natural environments, and that a higher DOC level is a major predictor of infected amoeba by amoeba-associated bacteria, such as *Legionella* species [29, 61]. Thus, factors controlling the release or uptake of DOC in the cooling tower environment may have important influences on the host cell population. In natural ecosystems, DOC uptake into the trophic chains is usually controlled by grazing activity, by the protozoan population and viral lysis [62, 63]. However, disinfection schedule is likely another important factor in cooling towers. The use of chlorine and other biocides will cause a certain amount of cellular death, and thus, release DOC into the system [64]. Thus, different effects may be observed for continuous versus periodic applications of disinfectant. Presumably, periodic application could release more DOC by generating peaks of cellular death, but this would require additional studies.

### Effect of DOC on cooling tower eukaryotic communities

In previous work, we observed that higher levels of DOC correlated with lower levels of bacterial alpha diversity, and that this lowered level of alpha diversity was associated with the dominance of *Pseudomonas* groups [2]. The presence of *Pseudomonas* groups has been shown to negatively correlate with the presence of *Legionella* [1, 2, 65, 66]. In contrast, our results indicated that eukaryotic diversity increased with higher levels of DOC. In the case of beta diversity, high and low DOC samples clustered distinctly, indicating distinct communities. A probable cause for this observation may be the introduction of “contaminating” organisms. By this, we mean the presence of eukaryotic organisms not naturally growing in natural or engineered water systems. Cooling towers intake great volumes of air due to their function and design [67]. This can lead to the presence of airborne fungal spores, fungal tissues, insects, plant tissues and seeds in cooling tower water. Therefore, we defined contaminating OTUs as OTUs belonging to the fungal groups *Ascomycota* and *Basidiomycota*, as well as, insects (*Arthropoda*) and plants (*Phragmoplastophyta*). Although yeast and moulds were identified, the majority of the fungal groups were associated with macroscopic fungi (mushrooms), usually found in forests. Furthermore, most of the *Phragmoplastophyta* OTUs were associated with land plants belonging to taxa comprising of grasses and trees. The Arthropods detected were mainly flying insects associated with the *Diptera* order (flies and mosquitos), but beetles (*Coleoptera*) were also identified. It is noteworthy that some of the species included in these groups have aquatic larval stages and might therefore be resident of the cooling towers. When we grouped the contaminating OTUs together and plotted the counts as a function of DOC, a modest positive correlation was observed between these two factors. It is noteworthy to mention that this correlation was mainly driven by the *Basidiomycota* group. Consequently, the results suggest that contaminating OTUs may have some effect on the concentration of DOC within the cooling tower environment, depending on the location of the cooling tower. In this perspective, cooling towers in rural areas or close to green space may receive much more contaminating OTUs than cooling towers in dense urban areas. Other factors surely contribute to DOC concentrations, such as disinfection strategies (as discussed above), source of makeup water, cooling tower design, as well as factors associated with wind, which would affect the presence of contaminating OTUs [67]. Our study suggests that the location and surroundings of a cooling tower may be important to consider when developing a management strategy.

### Network analysis and cooling tower ecology

Network analysis revealed co-occurrence patterns between different taxa in the cooling tower environment (Fig. 4). In our case, the connections between taxa are based on Pearson’s correlation. These correlations could be due to ecological interactions, such as competition or mutualism, or because they occupy the same niche. One of the main findings of the network analysis was that the cooling tower ecosystem was modular with the identification of three modules within the network. The property of modularity indicates that the network contains a specific number of modules, where a module is a group of taxa that interact mostly with the members of its own module and less with other taxa from other modules in the network [51]. One potential interpretation of the presence of these modules in the network is that they represent distinct sub-niches in the cooling tower ecosystems. However, modularity may also be due to the presence of several phylogenetically related nodes sharing the same optimal niches.

The identification of several connector nodes within the modules may indicate that several taxa may be able to inhabit various niches or that certain niches may overlap. Connector nodes are believed to represent generalist ecological behaviour [51, 53]. For instance, *Cytophaga* was a connector node identified in module 2. These bacterial species are known to be important consumers of various large organic compounds, such as cellulose and chitin, either through direct consumption of dissolved organic matter or by lysing species containing these compounds, such as cyanobacteria [68]. Correspondingly, we observed that this taxon was connected to several algal nodes from modules 2 and 3. Thus, *Cytophaga* may be able to inhabit various cooling tower niches containing algal organisms through direct nutritional interactions with these groups. On a side note, the capacity to degrade heavy weight organic compounds has made *Cytophaga* an important actor of the microbial loop in marine systems [68]. This may indicate that connector nodes are important keystone species, as they could potentially affect the members of several sub-niches.

### Microbiome of cooling tower and *Legionella* ecology

Our analysis revealed specific trophic interactions that might be important for *Legionella* ecology*.* This was clearly recognizable with the interaction between *Oligohymenophorea* and *Brevundimonas* identified by the network and LEfSe analyses. Our results showed that bacterial species belonging to the *Brevundimonas* genus could be used as predictors for identifying towers with high levels of *Oligohymenophorea* and *Legionella* [2]. The predator-prey interaction between *Tetrahymena* and *Brevundimonas* was confirmed in vitro (Fig. 6). In this scenario, *Brevundimonas* would be used as a food source for the growth of the ciliate community, which would grow in numbers. This would then allow *Legionella* species to grow in the cooling tower environment, as they would use these ciliates as host cells. In addition, *Oligohymenophorea* likely prey on other microorganisms identified with the network analysis and/or LEfSe, such as the bacteria *Sphingopyxis*, *Reyranella*, *Qipengyuania* or *Skermanella*, and the algae *Trebouxiophyceae* and *Chrysiophyceae*, identified with the network analysis. These taxa were previously found to be predictors of the presence of *Legionella* in cooling towers [2].

Additionally, our results demonstrated that *Brevundimonas* SPF441 could stimulate the growth of *L*. *pneumophila* on CYE agar without l-cysteine supplementation (Fig. 8). This stimulation may be due to the production of essential amino acids, and more specifically production of cysteine, by the *Brevundimonas* isolate. Indeed, whole genome sequencing identified several genes involved in the synthesis of cysteine or cysteine derivatives in *Brevundimonas* SPF441. Cystathionine gamma-lyase and cysteine-*S*-conjugate beta-lyase are both enzymes involved in production of thiocysteine and l-cysteine from l-cystine [69]. Cysteine from the yeast extract in CYE agar is quickly oxidized to l-cystine and cannot be used by *L*. *pneumophila*, hence the necessity to supplement the CYE medium with l-cysteine [70]. Consequently, *Brevundimonas* SPF441 may supplement *L*. *pneumophila* with an exogenous source of l-cysteine by converting the l-cystine to l-cysteine and thiocysteine. In addition, *L*. *pneumophila* is auxotrophic for several amino acids, such as arginine, cysteine, isoleucine, leucine, methionine, threonine and valine [71]. *Brevundimonas* SPF441 possesses all the genes necessary for the production of these amino acids. Thus, our results suggest that *Brevundimonas* species may be important for *Legionella* ecology, through direct and indirect interactions resulting in the promotion of *Legionella* survival and growth in water systems.

Overall, these results have several implications for *Legionella* ecology. First, the direct and indirect stimulation of *Legionella* by *Brevundimonas* seems to suggest that these two species participate in mutualistic interactions. In the context of a water system, an interesting possibility is that *Brevundimonas* stimulates the growth of *Legionella* in order to protect itself from predation by ciliates. Promoting the nearby survival or growth of *Legionella* may reduce the chance of *Brevundimonas* from being ingested by predators by reducing the number of predators, as *Legionella* will kill them. On the other hand, *Legionella* receives the benefit of a higher chance of survival and proliferation through nutritional supplementation (both through cysteine and host cells). Secondly, the *Brevundimonas*-*Legionella* interaction supports the idea that specific groups of organisms, other than host species, are crucial for *Legionella* ecology. Therefore, some specific microbiomes would be more permissive for colonization, survival and proliferation of *Legionella* than others. In this perspective, towers with more negatively interacting species would be less permissive (even refractory), whereas microbiomes with higher levels of positively interacting species would be more permissive towards *Legionella.* Nevertheless, understanding which microbiomes would be permissive or not would require additional studies. A potential starting point would be to examine the other positively interacting species from our dataset.

Taken together, our results suggest that ciliates species may be more important than previously thought for *Legionella* ecology, as most research mainly focuses on the free-living amoeba population [61, 72–74]. The importance of the ciliate community was recently suggested [31]. It is tempting to speculate that the ciliate population may increase the virulence of *L*. *pneumophila* similarly to what was previously demonstrated in the amoeba *A*. *castellanii* [75].

Finally, *Pseudomonas* is negatively associated with *Legionella* due to direct or indirect interactions [2]. Our results support the latter possibility, since *Pseudomonas* correlates negatively with *Oligohymenophorea* (Fig. 5b) and is a predictor of cooling towers free of *Oligohymenophorea* (Fig. 6). This could be due to direct killing, since *Pseudomonas aeruginosa* is able to kill amoeba using its type three secretion system [76]. This may also be true for some species of ciliates, but several *Pseudomonas* species have also been identified as prey for ciliates, such as *Tetrahymena* [77, 78]. Thus, more research should go into identifying the mechanisms that promote the presence of *Pseudomonas* in towers without ciliates.

## Conclusion

In conclusion, our research indicates that the host community is not the single most important factor for *Legionella* outbreaks, but that instead, *Legionella* ecology is dependent on various groups of microorganisms. As a result, this may indicate that the microbiomes of cooling towers, which may be more or less permissive for *Legionella*, is a crucial component in *Legionella* ecology. This permissiveness would be directly related to the species that are present in the microbiomes. As a consequence, our research indicates that *Legionella* proliferation in cooling tower rely on a network of organisms, many of which have yet to be characterized, representing a “dark web” of interactions. Complex microbial interactions between primary producers, bacterial heterotrophs as well as microbial grazers are seemingly important for *Legionella* ecology. More specifically, the ciliate community appears to be an important factor to consider for *Legionella* outbreaks in cooling towers. Furthermore, the role of carbon flux and microbial grazing in the microbial loop may have an important role in *Legionella* ecology. Finally, potential biomarkers for predicting the presence of *Legionella* and Ciliates were identified using LEfSe. *Brevundimonas* is an obvious candidate because of its positive relationship with both *Legionella* and ciliate hosts. It is warranted to examine the ecological roles of the bacterial biomarkers that were found to predict the presence of *Legionella* and the host taxa, and how they would come in to play in *Legionella* ecology. The manipulation of a cooling tower’s microbiome to create a non-permissive environment for the colonization by *Legionella* may be a way to reduce the number of outbreaks of Legionnaires’ disease. This could likely be achieved by adjusting operating parameters.

## Materials and methods

### Sampling of cooling towers and parameter measurements

A total of 18 cooling towers were sampled from six different regions in Quebec, Canada, between the 10th and 21st of July 2017. Details were presented in previous work [2]. Briefly, water was sampled in 1-L sterile bottles three times, from the water basin of cooling towers (Supplementary Table S3). The Biomass was collected by filtration (0.45 μm pores) and DNA was extracted using the DNeasy Power water kit from QIAGEN (Cat. No. 14900-100-NF) [2]. The *16S rRNA* gene targeted amplicon sequencing using the Illumina MiSeq platform (NCBI Sequence Read Archive accession number PRJNA507738) and the quantification of *L*. *pneumophila* using qPCR was reported previously [2].

### Eukaryotic community profiling of cooling towers

*18S rRNA* amplicon sequencing was performed using the Illumina MiSeq platform (Illumina, Inc.) as described in the Earth Microbiome Project’s (EMP) 18S Illumina Amplicon Protocol [58, 79, 80]. This protocol targets the V9 region of the *18S rRNA* gene by using primer 1391F (5′-GTA CAC ACC GCC CGT C-3′) and EukBR (5′-TGA TCC TTC TGC AGG TTC ACC TAC-3′) [79, 80]. The Illumina two-step indexing protocol was used, where the Illumina overhang adapters were added to the primers described above. The V9 hypervariable region of the *18S rRNA* gene was amplified by PCR, using the Paq5000 PCR Hotstart master mix (Agilent Technologies, California, USA) with 10 μM of each primer and 2 μl of DNA. The cycling program consisted of an initial denaturation step at 94 °C for 3 min, followed by 30 cycles of 94 °C for 45 s, 57 °C for 60 s and 72 °C for 90 s, and a final elongation step of 10 min at 72 °C. The size of the PCR products (260 ± 50 bp) was confirmed on a 2% agarose gel. The PCR products were purified using the Ampure XP bead kit (Beckman Coulter, Indianapolis, IN, USA) following the manufacturer’s instructions. The purified PCR products were then indexed using the Nextera XT indexing kit, according to the manufacturer’s instructions (Illumina, Inc.). The indexed PCR products were then purified using the Ampure XP bead kit and visualized on a 2% agarose gel. The purified DNA was quantified using the Quant-iT PicoGreen dsDNA assay kit (Thermofisher, MA, USA). The DNA samples were normalized to a concentration of 4 nM. The samples were pooled, diluted and denatured with NaOH to a final concentration of 20 pM in 1 mM NaOH and HT1 buffer (Illumina, Inc.). This solution was further diluted down to 4 pM in pre-chilled HT1 buffer. Following the same dilution protocol, 4 pM PhiX control (Illumina, Inc.) was produced. The solutions were combined in a microcentrifuge tube to produce a 15% PhiX spike in, with 90 μl of the PhiX solution and 510 μl DNA library. This solution was heat denatured for two minutes at 96 °C and then chilled on ice for 5 min. The sample (600 μl) was loaded in a Miseq platform using the 600 cycles MiSeq Reagent Kit v3.

Sequencing data was processed using the Mothur pipeline [81]. Briefly, the paired reads were assembled into contigs. Any contig with ambiguous bases or lengths exceeding 310 bp were culled. The sequences were aligned to the eukaryotic Silva Reference Database release 132. We customized the database so that it contained only the V9 region of *18S rRNA* genes. This provides better alignments and ensures that the reads overlap with the appropriate region of the database. Then, the ends and gaps from the sequence alignment were trimmed so that all sequences had the same alignment coordinates. The sequences were further denoised using a pre-cluster algorithm within Mothur. The resulting unique sequences were purged of chimeras using the VSEARCH algorithm implemented by Mothur. Additionally, any remaining undesirable sequences, such as sequences from Bacteria, Archaea, chloroplasts and mitochondria were removed by, first, classifying the sequences with Bayesian classifier algorithm within Mothur, and, then removing the undesirable sequences. Supplementary table S1 provide the number of sequences left after each key steps of the processing of the data. The sequences were then assigned de novo into OTUs using the cluster.split command with a cutoff of 0.03. The clustering created a total of 44 183 OTUs and a taxonomy file for each OTU. Two intermediate datafiles were created for different analyses or visualization purposes. The first one is an OTU table contained the OTU counts for each replicate (hereafter called table R1 for clarity). The second one was created by averaging the counts for the three replicates of each cooling tower sample (herafter called table A1 for clarity). The average OTU counts for table A1 were rounded down to the closest integer.

Table A1 was mainly used for ecological and statistical analysis, whereas table R1 was mainly used for visualization of each replicate (Fig. 1). Both tables were processed using the Microbiome Analyst [47], which performs data filtration and several ecological analyses, such as community profiling, clustering and biomarker analyses. The low count filter was set so that OTUs are retained only if at least 20% of their values contain at least 2 counts. This removed a total of 16,736 and 4835 low count OTUs from table R1 and table A1, respectively. Additionally, a default low variance filter, which removes OTU with low variance at 10% using inter-quantile range, was used to remove any OTUs that were constant throughout the samples. This removed a total of 75 and 80 OTUs from table R1 and A1, respectively. Next, the tables were rarefied to the minimum library size and total sum scaling was used to normalize the data. Table R1 contained a total of 2951 counts per replicate and table A1 contained a total of 3484 counts per sample. Furthermore, the raw reads of the *18S rRNA* amplicon sequencing have been uploaded to NCBI’s sequence read archive under the accession number PRJNA563440.

Additionally, a negative control was run separately to determine contamination levels. This blank consisted of running an unused sterile filter through the same pipeline as aforementioned. Thus, the clean filter was processed for DNA extraction, *18S rRNA* PCR amplification and sequencing on the MiSeq Illumina platform (V3 reagent kit, 600 cycles). The same mothur pipeline was used to process the raw reads.

### Ecological analysis

Next, the MicrobiomeAnalyst was used to create the taxonomic abundance profiles, the coverage analysis, the alpha and beta diversity analysis and the LEfSe analysis [47].

#### Good’s coverage estimator

Good’s coverage estimator was calculated for each sample using table A1. This was calculated on the unrarefied/unfiltered and rarefied/filtered eukaryotic OTU table (Supplemental Table S2).

#### Taxonomic abundance profiles

Table R1 was used to create the taxonomic profile of each replicate. MicrobiomeAnalyst was used to group the OTUs at the phylum level. The host taxa profile was created by creating a count table with the OTUs that were assigned to the genus *Acanthamoeba*, *Naegleria*, *Oligohymenophorea* and *Vermamoeba* for each replicate. The group “other” was created by subtracting the total counts of the host taxa from the total counts for each replicate. GraphPad prism version 8.3.1 for macOS was used to create the bar graphs and visualize the data.

#### Alpha diversity analysis

The Shannon index was calculated for each replicate using table R1. This was done with the microbiome analyst. The data was inputted into GraphPad prism version 8.3.1 for macOS for statistical testing and visualization. Spearman’ rank correlation was conducted on the averaged values of the three replicates for each tower. The average Shannon index of each sample was plotted with error bars showing standard deviation. A semi-log model best fitted the data for the regression analysis. We used a *P* value cut-off of 0.05 to assess statistical significance for both analyses.

#### Beta diversity analysis

Beta diversity was performed on both table A1 and R1. Briefly, the Bray-Curtis index was used to create a dissimilarity matrix for both OTU tables. Non-metric multidimensional scaling was used as an ordination method. The ordination results from table R1 were used for creating the NMDS plot. This was done with R and the Tidyverse package to visualize the dissimilarity between all replicates [82, 83]. ANOSIM statistical analysis was performed on the ordination results of table A1.

#### LEfSe analysis

The LEfSe analysis was performed on the bacterial profiles of the same cooling tower samples we had sequenced in a previous experiment [2]. The raw reads for this bacterial data set have been uploaded on NCBI Sequence Read Archive under the accession number PRJNA507738. Briefly, towers were categorized by the relative abundance of *Oligohymenophorea* (using table A1): absence (less than 0 read counts per sample), low level (between 1 and 100 read counts per sample) and high level (more than 100 read counts per sample). LEfSe analysis was performed on the resulting OTU table previously described (see [2]). This analysis was conducted through the microbiome analyst but GraphPad prism was used to create the bar plots and visualize the data. We used a *P* value cut-off of 0.05 for the Kruskal-Wallis test and the Wilcoxon test. The LDA score (log scale) cut-off was set at 3.

### Network construction and analysis

A network, based on co-occurrence, was constructed between the eukaryotic and bacterial taxa of the cooling towers using the Molecular Ecological Network Analysis pipeline (MENAp) [51]. Briefly, eukaryotic OTUs were regrouped into their respective families using OTU table A1. Bacterial OTUs from the previous study were grouped according to their respective genera [2]. OTUs belonging to the contaminating groups (*Ascomycota*, *Basidiomycota*, *Phragmoplastophyta* and *Arthropoda*) were removed from the eukaryotic dataset. Towers left with less than 75% of the initial number of sequences were removed. Therefore, only towers CN1, CN2, CN3, MTL1 to MTL6 and MTL8 were kept for this analysis. The eukaryotic data were rarefied to the sample with the least sequence count (8328 counts per sample). Similarly, the bacterial dataset was rarefied to the smallest sample (22,216 counts per sample). OTUs contributing for less than 0.1% of the total number of counts were merged together into a group called low count OTU. This was done separately for the bacterial dataset (low_count_Bacteria) and the eukaryotic dataset (low_count_Eukaryota). The two tables were merged and processed with the MENA pipeline via the following website: http://ieg2.ou.edu/MENA [51]. The network was constructed using the default settings of the pipeline with the exception of the following parameters: the “Majority” setting was set to 1, the “Logarithm” function was not used and Pearson correlation coefficient was selected to calculate correlations between different OTUs. MENA uses random matrix theory to identify a reliable Pearson’s correlation coefficient as a cut-off based on the χ^2^ test with Poisson distribution [51, 84]. In our case, MENA identified a Pearson coefficient of 0.32 as the cut-off when using the strictest threshold of χ^2^ > 0.05. Cytoscape 3.7.1 was used to visualize the network [52].

### Isolation of *Brevundimonas* sp. from cooling tower

Bacterial colonies were isolated from a cooling tower on R2A agar and re-streaked three times to ensure pure cultures. Glycerol stock (15% glycerol in R2A medium) cultures were made for each strain for downstream applications. The identities of morphologically different colonies were determined by sequencing the *16S rRNA* gene. Briefly, DNA was extracted from pure cultures using the Wizard genomic DNA purification Kit (Promega). The *16S rRNA* gene was amplified by PCR, using bacterial primers 27F (5′-AGAGTTTGATCMTGGCTCAG-3′) and 1492R (5′-TACGGYTACCTTGTTACGACTT-3′). The PCR product was cloned into the pGEM-T Easy vector system (Promega). Clones were selected by blue white screening. Plasmids containing the *16S rRNA* insert were extracted using a Miniprep plasmid extraction kit (QIAGEN). The insert was sequenced by Sanger sequencing at the Plateforme Génomique de l’Université Laval, Canada. The sequence was then analysed using NCBI BLAST. One of the isolates of interest showed 99.51% identity with *Brevundimonas* sp. strain HES1 (Accession MN081030.1). We named the strain *Brevundimonas* SPF441.

### Whole genome sequencing of *Brevundimonas* SPF441 isolate

Genomic DNA was extracted from the *Brevundimonas* SPF441 isolate using the Wizard genomic DNA purification kit (Promega). The genomic DNA quality was verified on a 0.8% agarose gel and the concentration was determined using the Quant-iT PicoGreen dsDNA assay kit (Thermofisher). The DNA library for whole genome sequencing was prepared using the Nextera XT DNA library prep kit (Illumina), according to the manufacturer’s instructions. The library was analysed on an Agilent Technology 2100 Bioanalyzer (Agilent) to evaluate proper DNA fragment size. The library was normalized to 2 nM and then pooled together. The pooled library was denatured with 0.2 N NaOH and diluted to 12 pM loading concentration with HT1 buffer as per the manufacturer’s instructions (Illumina). The library was spiked with PhiX control (20 pM) at 1%. The library was then loaded on the MiSeq sequencing platform (Illumina) with the MiSeq Reagent kit V3 (600 cycles).

A total of 500,485 paired reads were generated. The read quality was evaluated using FastQC [85]. The forward and reverse sequences were processed using Trimmomatic (v0.39) with the following commands: LEADING: 10 TRAILING: 10 SLIDINGWINDOW: 5: 20 MINLEN: 36 [86]. This removed low-quality reads, leaving 467,366 reads (93.38% of initial data). The forward and reverse reads were assembled using SPades (v3.13) [87]. The reads were first corrected using the “only-error-correction” option, and then the corrected reads were assembled using the “only-assembler” option. When assembling the reads, the k-mer length was set to 21, 33, 55, 77, 99 and 127. The assembled genome was uploaded to MiGA (Microbial Genome Atlas, v0.3.12) server, and the NCBI Prok module was used to identify the taxonomy and novelty of the isolate [55]. Bandage was used to infer the quality of the assembly [88]. Additionally, the assembled genome was annotated using Prokka (v1.14) [89], and uploaded to the blastKOALA (v2.2) website to infer metabolic pathways present in the isolate, using the KEGG database [90]. The raw reads of this genome were deposited to NCBI SRA under the Bioproject number PRJNA580507. The Whole Genome Shotgun project for *Brevundimonas* SPF441 has been deposited at DDBJ/ENA/GenBank under the accession number WJWX00000000. The version described in this paper is version WJWX01000000. This deposited genome was annotated using the NCBI Prokaryotic Genome Annotation Pipeline.

### Co-culture of *Brevundimonas* with *Tetrahymena*: evaluating the fate of *Brevundimonas* SPF441

The fate of *Brevundimonas* SPF441 when incubated in coculture with *Tetrahymena thermophila* and *Tetrahymena pyriformis* was determined by CFU counts. Briefly, *T*. *thermophila* and *T*. *pyriformis* were grown in SPP medium (Sugar Proteose Peptone: 8 g proteose peptone, 0.8 g dextrose, 0.4 g yeast extract and 33 nM FeCL, in 400 of distilled water) at 30 °C and 21 °C, respectively. Cells were passaged when the density reached 5 × 10^5^ cells/ml. Twenty-five millilitres of the cell cultures were transferred into 50 ml conical tubes and centrifuged at 600 *g* for 5 min. The supernatant was quickly removed and 25 ml of plate counting broth (PCB: 5 g yeast extract, 10 g tryptone, 2 g dextrose, 1 L water) was added to each tube. One-millilitre aliquots of each ciliate solution were transferred to six wells of two 24-well plates. Six wells on each plate were filled with 1 ml aliquots of sterile PCB to be used as controls. Each well was inoculated with 30 μl of a 0.4 OD_600 nm_ *Brevundimonas* SPF441 suspension, resulting in a final inoculum of 4 × 10^6^ CFU/ml. The co-culture with *T*. *thermophila* was incubated at 30 °C while the co-culture with *T*. *pyriformis* was incubated at 25 °C. CFUs were determined at 0, 2, 4 and 12 h of incubation on nutrient agar. The plates were incubated at 30 °C for 2 days.

### Co-culture of *Brevundimonas* with *Tetrahymena*: evaluating growth of *Tetrahymena* using *Brevundimonas* SPF441 as food source

The growth of *T*. *pyriformis* and *T*. *thermophila* was determined when incubated in Tris buffer and periodically fed with the *Brevundimonas* SPF441 isolate. Briefly, both ciliates were grown in SPP media as described above to a concentration of 1.0 × 10^6^ cells/ml. The cells were washed twice in 10 mM tris (pH 7.5). *Tetrahymena* cells were counted using a Guava easyCyte flow cytometer, using the FSC and SSC parameters. The cells were then diluted down to 1.0 × 10^3^ cells/ml for the co-culture and 1.0 × 10^4^ cells/ml for the control (ciliates alone), in 25 ml of 10 mM tris (pH 7.5) solution. Ciliate cultures were counted before inoculation on day 0 and incubated at 25 °C and 30 °C, for *T*. *pyriformis* and *T*. *thermophila* respectively. Every other day, starting on day 1 of incubation, 200 μl of 1.000 OD_600 nm_ of *Brevundimonas* SPF441 isolate culture, washed twice in 10 mM tris solution, was inoculated into the ciliate cultures. Ciliates counts were measured on days 0, 2, and 4 using a Guava easyCyte flow Cytometer.

### Stimulation of *Legionella pneumophila* growth on CYE without l-cysteine

The stimulation assay was based on Wadowsky and Yee (1983) with slight modification [91]. Briefly, 100 μl of 0.2 OD_600 nm_ (around a total of 10^7^ CFU) of *L*. *pneumophila* suspension in AYE was inoculated in 5 ml of soft agar (0.5% agar). The soft agar was overlaid on CYE without l-cysteine supplementation and on CYE with l-cysteine (control). The agar was left to solidify for 15 to 30 min, after which, 10 μl of *Brevundimonas* isolate, at 0.2 OD _600 nm_, was spotted in the middle of the plates. The plates were incubated at 30 °C for 4 days.

## Supplementary information

**Additional file 1: Table S1.** Read count for each replicate of every cooling tower sample at the different processing steps.

**Additional file 2: Table S2.** Good’s coverage estimator for unfiltered and filtered eukaryotic OTU table for each cooling tower sample.

**Additional file 3: Table S3.** Characteristics of Cooling Tower Samples.

**Additional file 4: Figure S1.** Principal Coordinate Analysis (PCoA) of cooling towers showing clustering of eukaryotic community according to DOC levels using ANOSIM to evaluate dissimilarity between communities (R = 0.817041, P < 0.001).

**Additional file 5: Document DS1.** Description of metabolic features in *Brevundimonas* SPF441.

## Data Availability

The datasets generated and analysed during the current study are available in the NCBI Sequence Read Archive under the accession PRJNA563440 (*18S rRNA* gene raw reads), and PRJNA507738 (*16S rRNA* gene raw reads). The *Brevundimonas* SPF441 genome was deposited at DDB/ENA/GenBank under the accession WJWX00000000. The version described in this paper is WJWX01000000. The raw reads for this genome were deposited in NCBI sequence read archive under the BioProject number PRJNA580507.
