# Supplementary material for: Unravelling the importance of the eukaryotic and bacterial communities and their relationship with Legionella spp. ecology in cooling towers: a complex network
Source: Microbiome. 2020 Nov 12;8:157. doi: 10.1186/s40168-020-00926-6 (PMC7664032; doi:10.1186/s40168-020-00926-6)
Supplement: Supplementary file 4 — Additional file 3: Table S3. Characteristics of Cooling Tower Samples. [file 40168_2020_926_MOESM3_ESM.pdf]

Table S3: Characteristics of Cooling Tower Samples

Tower name	Total Iron (µl/L)	Dissolved Organic Carbon (DOC, mg/L)	Disinfection schedule	Administrative regions	Source of water
CdQ1	11	12.5	Weekly	Centre du Québec	Nicolet River
CN1	387	12.6	Daily	Capitale Nationale	St-Lawrence river
CN2	427	12.6	Continuous	Capitale Nationale	St-Charles lake, St-Lawrence river
CN3	805	5.92	Daily	Capitale Nationale	St-Charles lake, St-Lawrence river
CN4	603	35.0	Weekly	Capitale Nationale	St-Charles lake, St-Lawrence river
Out1	970	37.4	Continuous	Outaouais	Ottawa river
Out2	715	25.3	Continuous	Outaouais	Ottawa river
MTL1	109	13.8	Continuous	Montréal	St-Lawrence river
MTL2	55	15.8	NA	Montréal	St-Lawrence river
MTL3	209	17.8	Continuous	Montréal	St-Lawrence river
MTL4	72	11.3	Continuous	Montréal	St-Lawrence river
MTL5	82	10.0	Weekly	Montréal	St-Lawrence river

MTL6	155	8.24	Daily	Montréal	St-Lawrence river
Est1	738	16.1	Weekly	Estrie	St-François river
Est2	1384	40.8	Weekly	Estrie	St-François river
Mont1	134	15.1	Continuous	Montérégie	St-Lawrence river
MTL7	182	14.2	Weekly	Montréal	St-Lawrence river
MTL8	14	15.1	Weekly	Montréal	St-Lawrence river
