# Supplementary figures and images for: Unravelling the importance of the eukaryotic and bacterial communities and their relationship with Legionella spp. ecology in cooling towers: a complex network
Source: Microbiome. 2020 Nov 12;8:157. doi: 10.1186/s40168-020-00926-6 (PMC7664032; doi:10.1186/s40168-020-00926-6)

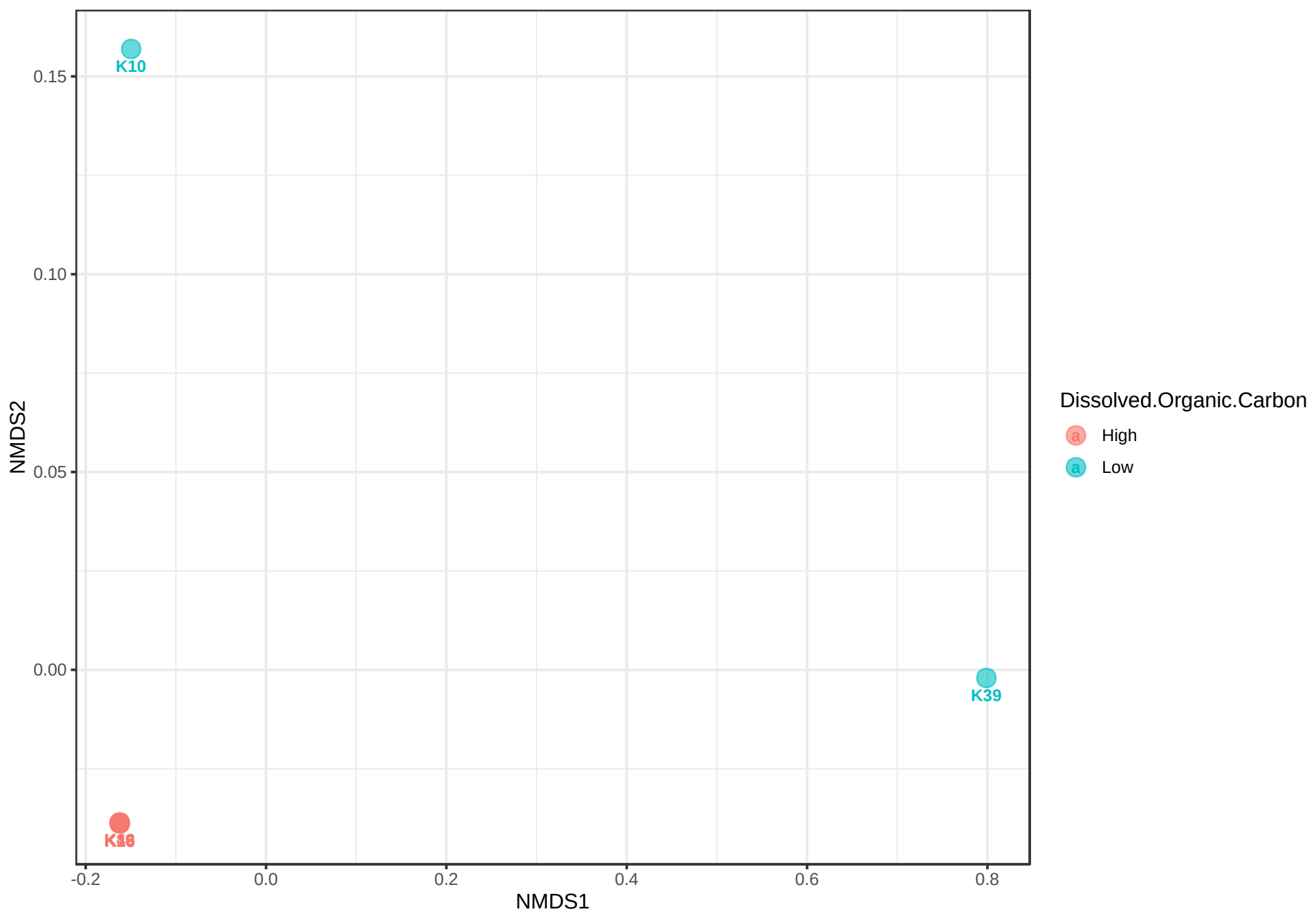

Supplement: Supplementary file 5 — Additional file 4: Figure S1. Principal Coordinate Analysis (PCoA) of cooling towers showing clustering of eukaryotic community according to DOC levels using ANOSIM to evaluate dissimilarity between communities (R = 0.817041, P < 0.001). [file 40168_2020_926_MOESM4_ESM.pdf]
