# Supplementary material for: Unravelling the importance of the eukaryotic and bacterial communities and their relationship with Legionella spp. ecology in cooling towers: a complex network
Source: Microbiome. 2020 Nov 12;8:157. doi: 10.1186/s40168-020-00926-6 (PMC7664032; doi:10.1186/s40168-020-00926-6)
Supplement: Supplementary file 6 — Additional file 5: Document DS1. Description of metabolic features in Brevundimonas SPF441. [file 40168_2020_926_MOESM5_ESM.pdf]

Document DS1: Description of metabolic features of Brevundimonas SPF441

Further analysis of the genome, using BlastKOALA, revealed that the *Brevundimonas* SPF441 isolate had 38% of its genes related to metabolism. This was shown by the number of different carbon metabolism cycles present in its genome. The carbohydrate metabolic pathways glycolysis, gluconeogenesis, the TCA cycle, the Pentose phosphate pathway, PRPP biosynthesis, the Entner-Doudoroff pathway, nucleotide sugar biosynthesis, Glyoxylate cycle, and propanoyl-CoA metabolism were all identified in this isolate. The genomic analysis also identified the presence of malate dehydrogenase, pyruvate orthophosphate dikinase, and phosphoenolpryuvate carboxylase (PEPC). These genes are usually associated with carbon fixation in plants, but can also be found in bacteria, such as *E. coli* [1]. PEPC is an enzyme that fixes bicarbonate with phosphoenolpyruvate to form oxaloacetate [1]. Thus, this may be important in several metabolic pathways. In addition, *Brevundimonas* SPF441 possesses the complete set of genes for biosynthesis of several amino acids, such as threonine, cysteine, methionine, valine, isoleucine, leucine, lysine, arginine, proline, histidine, and tryptophan. Genes for assimilatory sulphate reduction were also identified, which is an important pathway for incorporating sulphate molecules into different biological molecules, such as certain amino acids like cysteine [2].

References:

1. Smith TE. Escherichia coli phosphoenolpyruvate carboxylase: competitive regulation by acetyl-coenzyme A and aspartate. Archives of biochemistry and biophysics. 1970;137(2):512-22.
2. Rückert C. Sulfate reduction in microorganisms—recent advances and biotechnological applications. Current opinion in microbiology. 2016;33:140-6.
